# Cardiac synchrony, peer relationships, and affective experiences in children during group interactions

**DOI:** 10.1038/s41598-026-41275-y

**Published:** 2026-02-25

**Authors:** Bernadette F. Denk, Jens C. Pruessner, Stephanie Farah, Carmen Barth, Prasetia Utama Putra, Bigna Lenggenhager, Jeanine Grütter

**Affiliations:** 1 University of Konstanz, Konstanz, Germany; 2 Centre for the Advanced Study of Collective Behaviour, Konstanz, Germany; 3 AIR Association for Independent Research, Zurich, Switzerland; 4 Department of Psychology, University of Zurich, Zurich, Switzerland; 5 Ludwig-Maximilian-University, Munich, Germany

**Keywords:** Peer dynamics, Children, Physiological synchrony, Neuroscience, Physiology, Psychology, Psychology

## Abstract

The current study focused on the affective and physiological processes that may emerge during children’s dyadic peer interactions in groups. We hypothesized that physiological synchrony, the correspondence of physiological processes between individuals, would be associated with both the quality of the relationship and children’s individual and shared affective experiences. We tested this hypothesis in our sample of 187 children (mean age = 11.32, *SD* = 0.69), who arrived at the laboratory in groups of 4 to 6 individuals from the same classroom. During their interactions, we assessed all pairwise combinations within each group, resulting in 243 dyads with complete data. Before and after the group interaction, participants reported on their pre-established dyadic relationship quality with each group member (liking, closeness, and friendship), and reflected on their individual and shared affective experiences before the experiment and their affective experiences throughout the experiment using an Affect Grid measure. Participants listened to and discussed a story about the social exclusion of a peer. Heart rate was measured throughout the experiment using a one-channel ECG, and synchrony was calculated for each dyad using high-frequency (HF) and low-frequency (LF) cross-wavelet power. The results indicated increased LF synchrony in friends compared to non-friends. In contrast, dyads showed reduced HF synchrony when experiencing more positive affect. Exploratory results further suggested that synchrony may vary depending on different types of affective experiences for friends and non-friends. We discuss how physiological processes may be informative about the quality of short-term interactions and friendship patterns during peer group dynamics.

## Introduction

Positive peer group dynamics, such as positive interactions and relationships among peers, are a fundamental component of healthy emotional, social, moral, and cognitive development^1–3^. In contrast, experiences of negative peer group dynamics in childhood and adolescence can have negative long-term consequences on various outcomes, including self-esteem^4^, mental health^5^, academic engagement^6^, and academic achievement^7^. Since school is a primary context in which children interact with peers^8^, facilitating positive peer relationships in classrooms has been a main aim of interventions, especially during later childhood and early adolescence^9^. Although dyadic (i.e., pairwise) interactions are seen as a central building block for shaping the broader peer network, little is known about what characterizes dyadic social interactions and how specific social encounters contribute to longer-term peer dynamics^10^. Therefore, this study aimed to find out how dyadic social interactions during group activities relate to stable perceptions of relationships in older children’s friendship networks in their school class. In order to understand what happens when peers interact in groups, we focused on peer-to-peer interactions in a quasi-naturalistic setting and measured physiological interpersonal synchronization in the form of temporal alignment of children’s heart rate variability. This served as a marker of interpersonal connection, which we related to children’s dyadic subjective experiences and relationships. Interpersonal synchronization may work as a “social glue“^11^ that facilitates interpersonal exchange^12^.

The dynamics involved in interpersonal interactions can be characterized by multiple correlates, such as the content of verbal utterances exchanged by two people^10^, or the categorization of behaviors observed over time during an interaction^13^. One succinct, process-level measure of interpersonal interactions is interpersonal synchronization^14^, defined as the temporal correspondence between two or more individuals^15^. Synchronization can occur in different modalities: brain activity, physiology, movement, behaviors, and emotions, which possibly stem from common brain mechanisms^15,16^. Synchronization is believed to have evolved as a mechanism to facilitate connectedness and belonging in larger groups^17^, and is considered a crucial ingredient for social cohesion and cooperation^11,15,17^. *Physiological* synchrony, the temporal correspondence and interdependence between physiological processes^18,18,19^, offers a promising lens for studying peer interactions and their effect on social perception. Since peripheral physiological changes are closely related to emotional and social processes^20–22^, synchronization of markers such as heart rate may reflect shared emotional experiences and regulatory processes during peer interactions^23,24^. Because peripheral physiological data can be collected unobtrusively^25^, it enables the assessment of naturalistic interactions^26^.

Within physiological systems, changes in the activity of the autonomic nervous system are closely related to emotional and social functioning^22^, making autonomic synchrony a valuable marker of interpersonal dynamics^27,28^. The autonomic nervous system comprises the sympathetic and parasympathetic branches. The parasympathetic branch, commonly associated with relaxation, is also closely linked to emotion processing, including its social aspects^20^. Activation in the parasympathetic branch can be measured using heart rate variability (HRV)^29^. Increases in HRV have been linked to feelings of relaxation and contentment, while acute decreases have been linked to stress^20^. Synchrony in parasympathetic markers, such as HRV, could reveal dyadic interpersonal processes, such as emotional exchanges during interactions^30^. To assess HRV, parasympathetic activation can be measured in faster (high-frequency) changes in the heart rate trajectory. Slower, low-frequency changes represent a mix of sympathetic and parasympathetic influences^31,32^. To disentangle parasympathetic and sympathetic processes, synchrony can be measured for high-frequency and low-frequency processes separately, representing synchrony in the parasympathetic or sympathetic branches. Interest in physiological synchrony, including synchrony of the autonomic nervous system, is growing^18,33^. However, previous work involving children’s relationships mostly focused on interactions between infants and their caregivers. Multiple studies revealed that physiological synchrony may play an important role in how caregivers can support their infants’ emotional development^30,34–36^. Whether similar mechanisms operate in the peer relationships of older children is not yet well-understood, since we know little about to what extent autonomic synchrony relates to children’s peer social interactions. In order to study the conditions that give rise to synchrony in children’s peer interactions within groups, we investigated how relational and interaction-specific factors relate to physiological synchrony. Intending to clarify the processes shaping these interactions, we assumed that synchrony would reflect both stable social bonds and situational factors such as mutual attention or emotion regulation^12^.

We draw from theoretical frameworks that characterize peer experiences into different interconnected levels: the *individual* with their traits and other attributes, the *interactions* of multiple individuals, and the *relationships* between them (^37^; adapted from Rubin et al., 2006^38^). During social interactions, an individual’s behavioral tendencies interact with the tendencies of the peer, resulting in situation-specific behaviors (e.g., verbal expressions), emotions, and cognition. These dynamics are often embedded within long-term relationships characterized by mental representations of a person and the quality of the relationship with them, for example, as a friend. In late childhood and early adolescence, friendships – close, mutual, dyadic relationships – pose a particularly influential type of relationship. Friends interact differently from non-friends, typically displaying more smiling, cooperation, and sharing, and less competition^39,40^. Few studies have investigated autonomic nervous system synchrony in friends. In preschool-aged children, friends showed increased heart rate synchrony compared to non-friends^41^. In adults, the effect of relationship type and quality on synchrony was shown to depend on the interactional context, specifically on the task demands and affective valence of a situation^24,42–45^.

Beyond influences of the relationship, autonomic nervous system synchrony is linked to interactional affective processes: synchrony is particularly prevalent in moments when interaction partners exhibit *affect coherence* (i.e., the simultaneous experience of the same affect) or *emotional contagion* (i.e., the influence of one person’s affective state on another’s)^10,19,28,46^, and may reflect the ability to detect others’ affective states accurately (*empathic accuracy*)^26,47^. For example, during children’s group interactions, discussing a sad story may lead children to experience sadness simultaneously (affect coherence). In other cases, one child may feel sad, and other children may accurately perceive the sadness (empathic accuracy), accompanied by a change in their own mood towards sadness (emotional contagion). In each case, the processes may be facilitated by physiological synchrony^26^. In addition, the presence and degree of synchrony can vary depending on the affective tone of an interaction, for example, the positive or negative valence of a conversation^28,42^. Relational and interaction-specific affective factors also interact in predicting synchrony in a given situation. For example, in adults, good-quality relationships tend to be characterized by increased synchrony in positively valenced situations, whereas bad-quality relationships tend to elicit increased synchrony in negatively valenced situations^42–44^. Taken together, measuring autonomic synchrony during peer interactions may thus provide insights into the underlying emotional and relational dynamics shaping children’s social experiences.

**Fig. 1:**
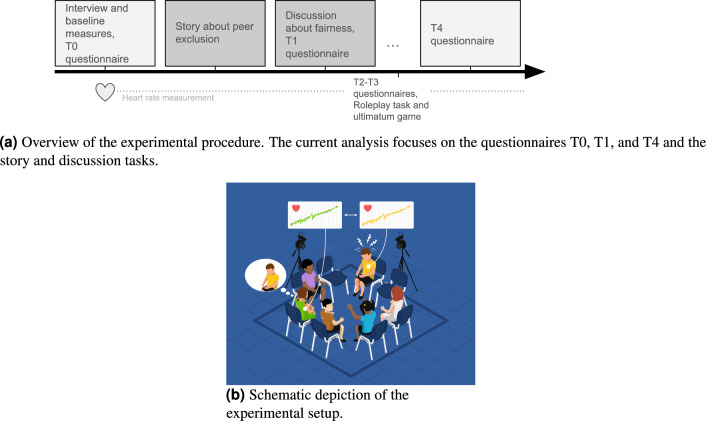
Overview of the study procedure.

In the present study, we investigated whether physiological synchrony in the autonomic nervous system, as measured by synchrony in heart rate, would be associated with the relational and interaction-specific affective characteristics of the dyadic relationship in children, specifically, the quality of the relationship and individual and shared affect. We investigated these associations in a quasi-naturalistic setting, where children explored socially and emotionally relevant topics, such as interpersonal fairness, exclusion, and friendship, in small groups (see Fig. 1). Children had known each other for at least one year, resulting in varying relationships within dyads. To confirm that different affective states that children may experience in this setting were associated with their physiology, our first hypothesis was that children’s individual HRV would be significantly lower than in a baseline activity and would significantly relate to children’s individual affect (valence and arousal). Moreover, we expected that individual HRV would be positively related to feelings of safety and contentment, and negatively with feelings of stress in the group. Our second – main – hypothesis was that autonomic physiological synchrony between two children would be related to a) their dyadic relationship quality, operationalized as the existing friendship status, as well as their situation-specific liking and closeness, and b) to the dyad’s affective interaction-specific experiences, operationalized as the total amount of affective valence and arousal, their affect coherence, and their empathic accuracy. We did not specify a direction for the hypothesis between dyadic relationship quality and autonomic synchrony, as previous work has yielded inconsistent results^24,41,48^. Regarding affective experiences, we had no directional hypotheses for affective valence and arousal, nor the similarity between children’s affective valence and arousal, as previous studies showed that both aversive and positive situations can produce synchronization^42,49^. We expected, however, that empathic accuracy, the ability to accurately recognize someone else’s affect, would be related to higher synchrony.

To better understand the autonomic processes involved in synchronization between peers, we chose cross-wavelet power as our analysis method^50,51^. Since this method captures a high temporal as well as high frequency resolution, we could specifically assess whether synchrony occurs in high or low frequencies and how it changes over time^51^. We thus investigated hypothesis 2 separately for high-frequency and low-frequency synchrony patterns. Other methodological approaches include the distinction between in-phase synchrony (i.e., trajectories changing in the same direction) and anti-phase synchrony (trajectories changing in opposite directions). We did not include this distinction here, but instead assessed the overall magnitude of shared autonomic activity in each frequency band.

## Methods

### Preregistration and ethic approval

The present study was preregistered after data collection but before data analysis. The preregistration can be retrieved at https://osf.io/tbrh9/?view_only=691e7e9eac814645a424a3ea0ac527ad (deviations from the preregistration are detailed in SM S3.4). This study has been reviewed and approved by the Institutional Review Board of the University of Konstanz (IRB 23/2023) and was conducted in accordance with the Declaration of Helsinki, specific regulations of the university, and relevant laws and regulations.

### Participants

Children from grades five to six (*N* = 187) between 10 and 13 years old (mean age = 11.32 years, SD = 0.69 years; 50.53% female) participated in the experiment. The study was part of a larger research project investigating the understanding of group dynamics in educational contexts. Out of 283 children that were invited for participation (all children of grades 5-6 in the participating school), 188 children and their primary caregivers gave their informed consent (66.43% consent rate), of which one child did not participate due to illness. Data collection took place in the summer of 2023, at the end of the academic year. In our study context, children transferred to secondary school in the 5th grade, and they participated in the experiment at the end of grade 5 or the end of grade 6. Therefore, the children in each group were classmates for at least one academic year. Participants were randomly assigned to a group with their classmates. Since we did not pre-select children for friendship status, the groups included a diverse set of relationships.

Out of *N* = 187 children, *n* = 16 children did not consent to wear a heart rate sensor. Additionally, *n* = 20 children had to be excluded from analyses related to heart rate due to bad data quality, resulting in a sample of *n* = 151 individuals whose data could be used to test hypothesis 1, related to individual data. However, due to missing data in questionnaire variables, the analysis included only *n* = 139 children. Out of *N* = 420 possible dyads, including all children, we obtained overlapping heart rate data for *n* = 243 dyads, whose data were used to test hypothesis 2, related to dyadic synchrony.

### Procedure

Figure 1a shows an overview of the procedure. 34 groups of 4 to 6 children (mean group size = 5.53, SD = 0.61) who are classmates came to the laboratory. The first part of the experiment consisted of a structured interview assessing dyadic relationship information and individual characteristics, as well as baseline measurements of physiological and questionnaire data. In each group, two to three children first completed the interview individually in separate rooms and later participated in the baseline measurement as a small group, while the other children completed the tasks in the opposite order. During the baseline measurement, children were familiarized with the heart rate sensors, and resting heart rate variability was measured. Additionally, children filled out questionnaires regarding their fellow group members, where they indicated feelings of closeness, liking, and perceived affect for each group member. When all group members had completed the baseline measurement and interview, they were seated in a circle in a room with video and audio recording equipment (see Fig. 1b). Here, the children were familiarized with the equipment and subsequently completed several group tasks. A main experimenter instructed the participants while two additional experimenters operated the technical equipment behind a partition. First, children listened to an audio recording of a story about peer relations and negative group dynamics at school. A structured discussion followed, during which the group collaboratively discussed questions related to the story. Two additional unrelated tasks followed, which were not designed to be part of the current study focus and will be discussed in different manuscripts. At the end of the experiment, participants answered additional questions about themselves and the other group members. Each participant received an educational children’s magazine for participation. Heart rate was measured continuously following the baseline measurement.

### Experimental tasks

#### Baseline heart rate measure

To measure heart rate and heart rate variability at rest, children were shown an emotionally neutral 5-minute video produced by us depicting various sections of the university where the experiment took place, accompanied by instrumental music and subtitles describing what was shown in the video.

#### Story about exclusion

Children listened to an audio recording of a story including transgressions within peer groups for 9 minutes. The story was developed with children and piloted before the experiment, as we aimed to create emotionally salient content with ambiguous characteristics. The main character, a girl, experiences social exclusion and bullying by her peers, and the negative consequences for her well-being are revealed in detail. Various other characters act as bullies, passive bystanders not helping the victim, or helpers intervening in the negative dynamic, whereby their thoughts and ambiguous reasons are highlighted. Some of the peers transition between these roles throughout the story. While listening to the story, children could look at the characters’ names written on sheets of paper. The story is described in detail in the Supplementary Materials (SM) S1.1.

#### Discussion task

Following the story, children were instructed to discuss several questions in a standardized procedure. The experimenter provided questions in writing and then retreated to leave the group to discuss among themselves. Ten questions were put in the middle of the seating circle successively, and the group indicated when their discussion of a specific question had ended. In some groups, not all ten questions were discussed due to limited time. The questions concerned the fairness of the various characters’ behavior and the characters’ emotions and reasoning (see SM S.1.2). The discussion lasted ten to fifteen minutes, depending on the group (for the analysis, the length was truncated, see *statistical analysis*).

### Measures

#### Physiological activity and physiological synchrony

*Heart rate* Heart rate was measured using the Polar H10 chest strap sensor (1000 Hz sampling rate; Polar Electro Oy, Kempele, Finland). Raw data R-R intervals were recorded using the HRV-Logger app for the IOS operating system^52^. R-R interval data were outlier-corrected using an in-house script by excluding values differing from the previous value by more than 30%^53^. Gaps of more than 2 seconds were interpolated using surrounding values. Individuals’ data were excluded when $\ge 10\%$ of the data were changed. Outlier-corrected R-R interval data were interpolated with a frequency of 1 Hz. We had preregistered to interpolate heart rate with a frequency of 4 Hz. However, we deviated from the preregistration by interpolating heart rate with a frequency of 1 Hz instead. This decision was made to achieve a more sparse representation of the data (instead of four data points per second, we used one data point per second for the analysis), which did not substantially change the temporal resolution of the heart rate data and revealed more easily interpretable findings. Heart rate was calculated in beats per minute [bpm]. Information about the preprocessing of heart rate data can be obtained in the SM S2.2.

*Heart rate variability* To quantify parasympathetic activation, we used the power in the high-frequency band (0.15–0.4 Hz) of Fourier-transformed heart rate signals (HF HRV), which is commonly used to assess parasympathetic processes^29,31^. HRV was calculated using the RHRV package in R^54^ for 120-second intervals with a shift of 60 seconds during the baseline measurement, during the story, and during the discussion.

*Heart rate synchrony* Synchrony between two children’s heart rate trajectories was calculated using an analysis based on wavelet transform, specifically, cross-wavelet power^50^. In this analysis, similarities between two wavelet-transformed trajectories (within each dyad) are quantified for each time point and for different frequency bands, resulting in cross-wavelet power coefficients ($\ge$0) for each time point and frequency band^55^. Cross-wavelet power has previously been used to quantify physiological synchronization^50,51,56^. Its advantage lies in a high temporal and frequency resolution, which allows for a detailed analysis of synchrony processes over time. Further, the method makes no assumptions regarding stationarity and linearity in the data, allowing for the analysis of synchrony in data, including oscillatory processes^55,57^.

Cross-wavelet power for each dyad was calculated using the WaveletComp package^55^. For high-frequency bands (0.125 to 0.5 Hz) and low-frequency bands (0.031 to 0.125 Hz), maximum coefficients were extracted for each 20-second interval (see^51^ and SM S2.2). This results in the measures *HF synchrony* and *LF synchrony* (high-frequency and low-frequency maximum cross-wavelet power). As the HF synchrony spectrum is associated with frequency changes in heart rate due to *parasympathetic* influences^20,31,32,58^, HF synchrony can thus be interpreted as synchrony in parasympathetic processes. Conversely, LF heart rate synchrony is associated with both sympathetic and parasympathetic changes^31^. Cross-wavelet power time series were calculated for the story and the discussion. Resulting values were z-scored such that the mean across all tasks and participants is 0 and the standard deviation is 1. This enables an easier interpretation of cross-wavelet power coefficients. Specifically, negative values indicate lower-than-average synchronization, whereas positive values indicate larger-than-average synchronization compared to other dyads. Large cross-wavelet power coefficients indicate overall synchrony. Specifically, we made no distinction between in-phase synchrony (i.e., a dyad’s values change in the same direction) or anti-phase synchrony (i.e., a dyad’s values change in opposite directions). Therefore, large cross-correlation results can indicate both types of synchrony. To account for intergroup variation in the duration of each task, synchrony time series were shortened to 8 minutes for the story and 10 minutes for the discussion in all groups. Additional details regarding the cross-wavelet power analysis can be obtained in SM S2.2.

#### Relationship quality

To assess the association between relationship quality and physiological synchrony, we measured both the more stable relationship type (i.e., friendship status) as well as the more variable situation-specific relationship quality (i.e., feelings of closeness and likability).

*Friendship* Dyadic friendship status was assessed during the interview (T0), when the children provided the names of all classmates they considered friends. The mutual friendship status (friendship, no friendship, or one-sided friendship) was determined by assessing whether both dyad members nominated each other as friends, only one member did, or neither did^59,60^. The average number of mutual friendships within each group was 28.06% (SD = 24.04%; range 0-100%). The average number of one-sided friendships was 14.20% (SD = 13.14%; range 0-33.33%).

*Closeness* We measured feelings of closeness using an adapted version of the Inclusion of the Other in the Self (IOS) scale^61,62^. The IOS is a one-item assessment where more or less overlapping circles represent closeness between a circle representing oneself and a circle representing the other person. Children rated their closeness to each other group member individually on a range from 0 to 6 at the beginning of the experiment (T0), after the discussion task (T1), and after one subsequent task (T2), whereby higher values reveal more closeness. Here, we included an averaged score across the measures at the beginning of the experiment and after the discussion task (T0 and T1; see SM S3.2)

*Likability* To assess liking in dyads, each child rated each group member regarding eight characteristics. Children rated how much they agreed that each group member was nice, fair, interesting, smart, cool, ready to help, boring, and mean on a 4-point Likert scale, whereby the negatively framed items were recoded (adapted from^63^). All eight items were assessed before and after the experiment (T0 and T4). The eight items showed good internal consistency (Cronbach’s alpha > .8) and a high correlation between before and after the experiment. Thus, we used averaged values taking into account liking before and after the experiment (T0 and T4; see SM S3.2).

#### Affective experiences

To assess the association of affective experiences with individuals’ HF HRV and with dyadic heart rate synchrony, we measured individuals’ affective valence, arousal, and experiences in the group, affect coherence, and empathic accuracy.

**Individual and Dyadic Affect** Affect was measured using the Affect Grid, a one-item scale consisting of a grid with two dimensions: valence and arousal^64^. We used a simplified version of the Affect Grid consisting of 3 x 3 fields (instead of the original 9 x 9), with both valence and arousal values ranging from 1 to 3. Horizontal values indicated valence ranging from “bad“ to “good“, while vertical values indicated arousal, ranging from “unexcited“ to “excited“. Participants indicated their affect after the discussion (T1), and each subsequent task (T2–T4), and estimated each of their group members’ affect during the baseline questionnaire (T0), after the discussion (T1), and after each subsequent task (T2–T4). Before the first use of the Affect Grid, the scale was explained to the participants using everyday examples. To ensure the participants’ understanding of the scale, control questions were asked, e.g., “What would you tick if you had an exam at school and why?”. Affect grid values at T1 were used as indicators for individuals’ affect.

From Affect Grid values, we calculated affect coherence between two group members’ Affect Grid “coordinates“, as

1 $$\begin{aligned} \text {affect coherence}_{ab} = \text {dist}_{max}-\sqrt{(\text {valence}_a-\text {valence}_b)^2 + (\text {arousal}_a - \text {arousal}_b)^2} \end{aligned}$$,

where $dist_{max}$ is the maximal possible distance $\sqrt{(3-1)^2+(3-1)^2} \approx 2.83$, and *a* and *b* denote two children from the same group. Affective coherence thus represents the closeness of the affective state between two individuals, and is re-coded such that higher values correspond to increased closeness.

Empathic accuracy, i.e., how close children’s estimates of others’ affect were to the other’s actual affect, was calculated similarly to affective coherence (Eq. 2).

2 $$\begin{aligned} \text {empathic accuracy}_{a} = \text {dist}_{max}-\sqrt{(\text {valence}_{est}-\text {valence}_{real})^2 + (\text {arousal}_{test}-\text {arousal}_{real})^2} \end{aligned}$$

This quantifies the distance between the estimated and real affective states, recoded such that greater values represent increased empathic accuracy. A similar approach had been used previously^47^ to quantify the distance between two persons’ two-dimensional data. In our case, we quantify the distance between two children’s affect data with the dimensions valence and arousal, obtaining a single value for each comparison.

In hypothesis 1, i.e., the relationship between HF HRV and affect, we used individuals’ T1 Affect Grid values. To test hypothesis 2, i.e., the connection between dyadic affect and synchrony, we included individuals’ T1 Affect Grid values (valence and arousal), as well as affect coherence, and empathic accuracy. Additional information about the values extracted from the Affect Grid can be obtained in SM S2.1.

**Group-related affect** In addition to subjective affect, we asked participants to what degree they felt safe, content, and stressed in the group, following the discussion (T1), one subsequent task (T2), and at the end of the experiment (T4). Participants answered on a four-point Likert scale ranging from “not at all“ to “very much“. We investigated the measurement following the discussion (adapted from^65^).

#### Covariates

In the analysis of hypothesis 1, which states that HF HRV would be influenced by valence and arousal, and group-related affect variables, we controlled for variables that are known to influence HRV measures, specifically body mass index (BMI; weight in kilograms divided by height squared in meters), age, and gender^58,66,67^.

When analyzing hypothesis 2, which states that relationship quality and affective experiences would influence the degree of heart rate synchrony, we considered the covariates gender combination and seating arrangement. Gender is associated with dyadic relationship characteristics^68^, and could thus be related to synchrony. Seating arrangement was based on the position that children had in the circle, and contained the levels “next to each other“, “across from each other“, and “not next to each other“ (i.e., another child between the two children in question).

For each hypothesis, individual connections between each potential covariate and the dependent variable were first investigated. The covariate was only included in the model when there was a significant relationship.

**Table 1:** Sample characteristics including dyads (*n* = 243) used in synchrony analysis.

Variable	*Mean* ±*SD* or %	Possible range
Gender combination	Female & Male: 43.62% Female & Female: 25.93% Male & Male: 30.45%	Female, male, or diverse
Friendship status	No: 55.97% Mutual friendship: 28.40% One-sided: 15.64%	No friendship, mutual friendship, one-sided friendship
Closeness sum	4.5 ±3.30	0–12
Liking sum	6.14 ±0.95	2–8
Affect coherence T1	1.71 ±0.76	0–2.83
Affective arousal T1 sum	93.89 ±1.12	2–6
Affective valence T1 sum	5.44 ±0.73	2–6
Empathic accuracy T1 sum	3.46 ±1.24	0–5.66

### Statistical analysis

All analyses were calculated in R^69^. Across hypotheses, multilevel models were created using the nlme package, and tested for significance as provided by the “summary“ function (see the nlme package documentation^70^). $R^2$ measures for fixed effects were calculated using the r2glmm package^71^.

#### Heart rate variability and affective experiences

To assess the association between HRV and subjective affective experiences, we employed a multilevel linear regression model using the package nlme^70^. The repeated HF HRV measures were nested within phases of the experiment, which were nested within the individual and within groups, warranting the multilevel model structure. Here, we followed the process described by Bliese & Polyhard (2002)^72^, which is described in detail in SM S3.1. The resting baseline served as the reference point for experiment phases in the model.

The intra-class coefficient (ICC) at the level of the child was 61.96%, indicating the clustering of HF HRV values within an individual^72^. The ICC at the level of the group was 15.75%, and group was included as a random effect in the model. A stepwise model comparison led to a model structure including random intercepts per child and group, fixed effects for time (within each phase), random slopes for time within each phase, and an autocorrelative structure. To assess the association between HRV and affective states, we added affect and the group climate measures “feeling safe“, “feeling content“, and “feeling stressed“ in relation to the group, which were measured after the discussion.

#### Heart rate synchrony, relationships, and affective experiences

To test our hypothesis that heart rate synchrony would be related to the relationship quality, as well as to affective experiences during the situation, we conducted multilevel linear regression models. To account for the nested structure of the data (repeated measures within dyads within groups), we included random effects for dyads and groups as described in Bliese & Polyhart (2002)^72^. Models were calculated for HF synchrony and LF synchrony separately. We compared a null model stepwise to more complex models via likelihood ratio testing (see SM S3.1). To assess the effect of time, we included repeated measures (assessed in 20-second segments) and the experimental phase. For the variable phase, the discussion phase was compared to the reference story phase.

For HF synchrony, the intra-class coefficient (ICC; see S3.1) was 15.26% for dyads, and 8.90% for groups. The final model included random intercepts for dyad and group, fixed effects for the experimental phase, a random slope for time within each phase, and an autocorrelative structure.

ICCs for LF synchrony were 7.75% for dyads and 2.13% for groups. The model to predict LF synchrony included random intercepts per dyad and group, a fixed effect for time within phase, a fixed effect for phase, and an autocorrelative structure (see SM S3.1).

To test the association between HF and LF synchrony with relational and affective characteristics, we included the independent variables friendship status, the sum of perceived closeness, the sum of liking, affect coherence, the sum of affective arousal, the sum of affective valence, and empathic accuracy. Some independent variables, such as feelings of closeness, were not shared between dyad members, as each child provided their own assessment of the other child. For these variables, we used the sum scores of both dyad members’ values to reflect the total expression of the variable in the dyad. Descriptive statistics for variables used as independent variables in this analysis are shown in Table 1.

## Results

### Heart rate variability and affective experiences

Our first hypothesis, i.e., the relationship between high-frequency (HF) HRV and affective characteristics in individuals, was tested using multilevel mixed models. HF HRV was measured during a resting baseline, a story-listening task, and a discussion task. Age and BMI were significantly related to HF HRV ($r_{age}$ = -0.05, *p* = .007; $r_{BMI}$ = -0.30, $p <.001$), and were thus used as covariates in the model. Gender was not related to HF HRV (*t*(2827.8) = -1.22, *p* = .221).

The regression coefficients, confidence intervals, and marginal $R^2$ are depicted in Table 2. The results show that HF HRV was lower during the discussion compared to the baseline. While the control variable age was not significantly related to HF HRV in the final model, a higher BMI was associated with decreased HF HRV. Increased HF HRV during the experiment was related to a more positive affect after the discussion. Moreover, children with higher HF HRV also showed reduced affective arousal. However, HF HRV was unrelated to contentment with the group, group-related feelings of safety, and group-related stress in our sample. Thus, hypothesis 1 was only partially supported. All fixed effects could explain 21.5% of the variance in HF HRV (95% Confidence Interval (CI) = [19.1, 24.4]). The full model explained all the variance at the level of the individual and 49.75% variance at the level of the group when compared to a model with only random intercepts.

**Table 2:** Regression coefficients for a multilevel model with the dependent variable HF HRV, random intercepts, and random slopes. * indicates $p<0.05$. The variable “Task” was dummy-coded with the resting baseline as a reference condition.

Variable	Estimate	95% CI [lower limit, upper limit]	*p*-value
(Intercept)	5.50	[5.31,5.69]	$<.001$*
Task: story	−0.03	[−0.12,0.07]	.571
Task: discussion	−0.10	[−0.2,0]	.041*
Time	−0.00	[−0,−0]	.012*
Age	0.07	[−0.04,0.17]	.199
BMI	−0.07	[−0.09,−0.05]	$<.001$*
Affective arousal T1	−0.13	[−0.22,−0.04]	.004*
Affective valence T1	0.34	[0.23,0.45]	$<.001$*
Contentment with group	0.07	[−0.05,0.19]	.270
Feeling safe in group	0.08	[−0.04,0.21]	.199
Feeling stressed in group	0.02	[−0.06,0.1]	.568
total $R^2$ 16.5%			

### Heart rate synchrony, relationships, and affective experiences

**Fig. 2:**
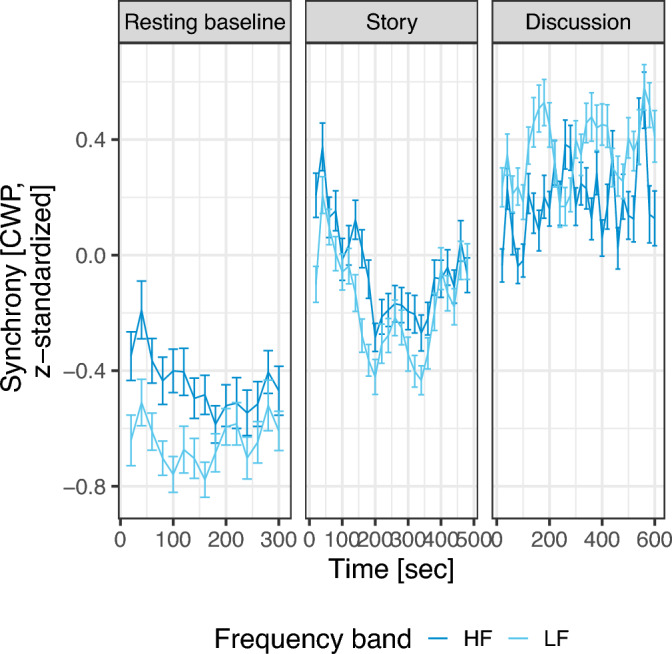
Average cross-wavelet power (CWP) trajectories in the sample, divided into high (HF) and low frequency (LF) bands, shown by experimental task. Resulting values are z-scored across all participants and tasks. Negative values represent smaller-than-average synchronization, whereas positive values represent larger-than-average synchronization. Synchrony values during the resting baseline are included for illustrative purposes for *n* = 118 dyads who completed the baseline simultaneously (in the analysis, only synchrony during story and discussion was analyzed).

The pairwise correlations between these variables are included in SM S4.1. The trajectories of HF and LF synchrony over time are depicted in Fig. 2.

We used multilevel models to test the association between HF and LF synchrony, respectively, and relational and affective characteristics of the dyad and interaction. Synchrony was measured during the story-listening task and the discussion task. Both HF and LF synchrony were significantly related to gender in a dyad (HF synchrony: *F*(2,10723) = 17.85, $p <.001$; LF synchrony: *F*(2,10723) = 11.09, $p <.001$), but not to seating arrangement (HF synchrony: *F*(2,10723) = 0.42, *p* = .654; LF synchrony: *F*(2,10723) = 0.57, *p* = .567). Gender combination was thus included as a covariate in the models. The model notations for HF and LF synchrony are detailed in the SM S3.3.

The model for HF synchrony showed that synchrony increased during the discussion compared to the story. We found decreased synchrony in all-female dyads compared to the reference group, mixed-gender dyads. All-male dyads did not significantly differ from mixed-gender dyads in HF synchrony. The model results indicate no effect of mutual friendship or one-sided friendship on HF synchrony compared to the reference group non-friend dyads. Closeness and liking were not significantly related to HF synchronization. Of the affect-related variables, the sum of positive affect in the dyad was related to HF synchrony, with a more positive affective valence after the discussion associated with lower HF synchrony during the experiment. Other affect-related factors, i.e., affect coherence, the sum of affective arousal, and empathic accuracy, yielded non-significant results. Effect sizes are shown in Table 3 (top). The full model explained all the variance at the dyadic level but no variance at the group level when compared to a model with only random intercepts.

**Fig. 3:**
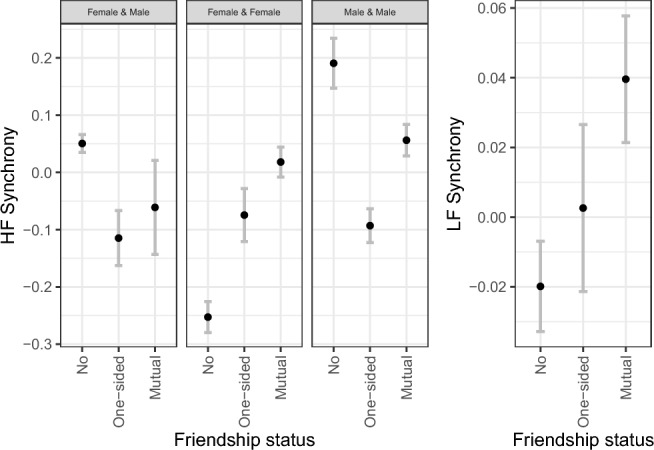
High-frequency maximum cross-wavelet power (HF synchrony) and low-frequency cross-wavelet power (LF synchrony) by friendship status. HF synchrony is additionally shown by gender combination to account for the interaction between gender and friendship status on HF synchrony. Synchrony values were z-standardized across all participants and tasks. Negative values represent smaller-than-average synchronization, whereas positive values represent larger-than-average synchronization. Mean and standard errors are shown.

LF synchrony increased during the discussion compared to the story. Within each task, there was no change in LF synchrony over time. All-female, but not all-male, dyads showed decreased LF synchrony compared to mixed-gender dyads. For relationship quality-related variables, we found significantly higher LF synchrony in mutual friendship dyads, but not one-sided friendships, compared to the reference group non-friend dyads (see Fig. 3, right side). Closeness and liking were not significantly related to LF synchrony outcomes. However, friendship status and closeness showed multicollinearity (see SM S4.1 and S4.2). When excluding closeness from the model, the effect of mutual friendship remained significant (*b* = 0.17; *p* = .006). When excluding friendship status from the model, the effect of closeness, likewise, remained nonsignificant (*b* = 0.00, *p* = .794). For affective experiences, there was no significant relation between LF synchrony and affect coherence, affective arousal, affective valence, nor empathic accuracy (see Table 3, bottom). The full model explained 82.69% variance at the level of the dyad and 54.26% variance at the level of the group when compared to a random-intercept model. For information about assumptions testing and model fit for both models, see SM S4.2.

**Table 3:** Regression coefficients, 95% confidence intervals, *p*-values, and marginal $R^2$ for HF synchrony and LF synchrony models. The variable “Task” was dummy-coded with story phase as a reference condition. “Gender” was dummy-coded and used the reference condition “male & female”. “Friendship” was added as a dummy-coded variable with the reference condition “no friendship”.

HF synchrony			
Variable	Estimate	95% CI [lower limit, upper limit]	*p*-value
(Intercept)	−0.07	[−0.17,0.04]	.207
Task: discussion	0.22	[0.17,0.27]	$<.001$*
Gender: female & female	−0.19	[−0.34,−0.04]	.013*
Gender: male & male	0.02	[−0.13,0.17]	.821
One−sided friendship	−0.10	[−0.26,0.07]	.246
Mutual friendship	0.11	[−0.07,0.3]	.226
Closeness (sum)	−0.01	[−0.04,0.02]	.594
Liking (sum)	0.02	[−0.06,0.11]	.566
Affect coherence	0.04	[−0.07,0.15]	.439
Affective arousal (sum)	0.02	[−0.03,0.06]	.426
Affective valence (sum)	−0.09	[−0.17,−0.02]	.018*
Empathic accuracy	−0.02	[−0.08,0.05]	.632
Total $R^2$ 3.2%			
LF synchrony			
Variable	Estimate	95% CI [lower limit, upper limit]	*p*-value
(Intercept)	−0.33	[−0.42,−0.23]	$<.001$*
Task: discussion	0.50	[0.43,0.56]	$<.001$*
Time	−0.00	[−0.00, 0.00]	.074
Gender: female & female	−0.14	[−0.25,−0.02]	.026*
Gender: male & male	−0.02	[−0.14,0.1]	.711
One−sided friendship	0.07	[−0.06,0.2]	.286
Mutual friendship	0.24	[0.09,0.38]	.001*
Closeness (sum)	−0.02	[−0.05,0]	.098
Liking (sum)	0.00	[−0.06,0.07]	.882
Affect coherence	0.00	[−0.08,0.09]	.975
Affective arousal (sum)	−0.01	[−0.05,0.02]	.535
Affective valence (sum)	0.03	[−0.03,0.09]	.280
Empathic accuracy	−0.03	[−0.08,0.03]	.318
Rotal $R^2$ 11.5%			

We conducted exploratory analyses to investigate potential interaction effects between friendship status (no friendship, one-sided friendship, or mutual friendship) and other variables included in the previous models: closeness, liking, affect coherence, affective arousal, affective valence, empathic accuracy, and gender combination. The results are shown in detail in SM S4.3. For HF synchrony, the model included significant interaction terms for friendship status and gender, where one-sided and mutual friendships were associated with higher HF synchrony for all-female dyads, but not for all-male dyads (all-female dyads x one-sided friendship: $\beta$ = 0.61, *p* = .032; all-female dyads x mutual friendship: $\beta$ = 0.53, *p* = .027). HF synchrony values by friendship status and gender combination in the dyad are depicted in Fig. 3 (left side). The interactions of friendship status with affect coherence, affective arousal, and affective valence did not reach significance (all $p >.05$).

An exploratory interaction model for LF synchrony analogously included interaction effects between friendship status and relational and affective variables. Here, affect coherence was related to lower LF synchrony in friends ($\beta$ = -0.27, *p* = .015), but did not significantly relate to LF synchrony within non-friend or one-sided friendship dyads. In addition, dyads with one-sided friendships (but not other friendship types) showed increased LF synchrony when the dyad experienced more affective arousal ($\beta$ = 0.15, *p* = .023). The interactions between friendship status and affective valence, or between friendship status and empathic accuracy, were non-significant (all $p >.05$).

## Discussion

Despite the importance of peer group dynamics in late childhood and early adolescence, the unfolding of interpersonal dynamics during peer interactions is poorly understood^10^. We assumed that interpersonal synchrony in peer interactions could elucidate these underlying processes^12^, and focused on interpersonal synchrony in the autonomic nervous system as a suitable marker to uncover dyadic emotional and social dynamics^21,30,44^. In our preregistered study, we examined how autonomic nervous system synchrony among 10- to 13-year-olds related to relationship quality and affective characteristics during peer interactions in small groups. We recorded heart rate synchrony during an educational setting, whereby participants listened to a story about social exclusion and discussed issues of fairness and morality related to the story. Children were recruited from the same classrooms and conducted these educational tasks in smaller peer groups of 4-6 participants.

To assess whether the individual physiological response was related to how individuals felt during the interaction, we examined HF HRV, an index of physiological relaxation (as a proof of concept; Hypothesis 1)^73^. HF HRV was significantly lower during the discussion as compared to the baseline, indicating increased physiological arousal. Moreover, higher HF HRV was significantly associated with a more positive affect and lower emotional arousal, underscoring the link between physiology and positive affective experiences during social interactions^20^. However, in contrast to our assumption, group-related affective variables showed no connection to HF HRV levels in our sample. Interestingly, the group itself explained about 15% of the variance in HF HRV values, potentially reflecting similarities to the task within the group (relative to overall differences between individuals). It is possible that individuals within groups were more similar in how they perceived the task and reacted to the tasks, not only at the physiological level, but also with regard to their motor behavior. Future work may more closely investigate how such group processes could predict fluctuations in individual HRV, particularly for those who align with perceptions of the group and those who do not. For example, group influences on HRV may be stronger for those who align with group members’ perceptions of the group than for those who do not. Alternatively, HF HRV may be more similar for individuals in groups who display similar motor behavior.

The main aim of this study was to go beyond the individual and examine how dyadic relational and interaction-specific characteristics shape dyadic interactions within groups. We assumed that the degree of interpersonal synchrony would be a marker for children’s relationship quality and their shared affective experiences within their peer interactions (Hypothesis 2). Through our analysis method, we were able to differentiate between LF and HF synchrony, which may reflect different underlying synchronization processes. While high-frequency processes in heart rate are markers of parasympathetic activation, low-frequency changes are shaped by both parasympathetic and sympathetic processes^31,32,58^. Across both tasks, friends showed higher LF synchrony, but not HF synchrony, than non-friends. Other characteristics of relationship quality, however, such as closeness and liking, were not significantly related to HF or LF synchrony. With regards to individual and shared affective characteristics, dyads who experienced more negative affect during the tasks showed increased HF, but not LF, synchrony. Taken together, only some aspects of children’s dyadic relationship quality and shared affective experiences were related to dyadic physiological measures, partially supporting hypothesis 2.

Friendships play an important role in human development^2^. However, there is limited previous work regarding friendship and synchrony with different age groups in different interactional contexts^24,28,41^. These studies observed increased cardiac synchrony within friendship dyads in preschool children during play situations^41^. For young adults, previous work identified increased synchrony within friendship dyads during positively valenced interactions^28^ and decreased synchrony in high-quality friendships during conflict situations^24^. The current study adds to this scarce previous evidence by highlighting that friendship status in late childhood and early adolescence may be connected to dyadic physiological processes in educational group settings, where children discuss emotionally salient topics. In our study, we saw that LF synchrony was positively associated with friendship status, as friend dyads showed more LF synchrony than non-friend dyads. Enhanced correspondence of LF heart rate activity between friends may indicate a shared response to emotionally arousing events^74^. Since this study cannot provide information about the mechanism behind the association of physiological synchrony and friendship, future work could shed more light on sympathetic processes and their association with emotion regulation during interactions of peers with different relationships (for example, with different sympathetic markers such as pre-ejection period or skin conductance^19,75^).

In addition to friendship, we investigated associations between children’s synchronization patterns and the emotional context of the interaction^28,42,43^. Children’s self-reported experiences of negative affect within the dyad were significantly related to increased dyadic HF synchrony. This type of synchrony reflects parasympathetic activation and plays an important role in emotional processing^30^. While this study cannot reveal the specific emotional processes that facilitated children’s HF synchrony, the correlation between negative affect and HF synchrony may reflect negative affect contagion. For example, if both dyad members responded with affective empathy towards the main character’s negative experiences, they may have synchronized more at times when they were emotionally affected by the story’s events. Moreover, synchronization processes may also facilitate children’s sharing of emotions, which may in turn result in feeling more negatively^26^. However, despite the connection between affect and HF synchrony, neither empathic accuracy nor affect coherence was related to synchrony. The affective response itself, rather than the relation to the other person’s affective response, may thus drive synchrony. Interestingly, while individual parasympathetic activation, as measured by HF HRV, was related to a more positive affect, parasympathetic synchronization within dyads was related to a more negative collective affect, suggesting that individual and dyadic processes can diverge in their associations^76^. Therefore, increased synchrony is not necessarily the result of increased absolute individual activation, but of corresponding changes between individuals of a dyad in the activation over time.

The connection between HF synchrony and affective states could explain why HF synchrony was related to friendship status only for all-female dyads. Girls may be more selectively influenced by others’ emotional states, such that HF synchrony only emerges with friends. In contrast, boys may synchronize parasympathetic processes independently of friendship status, with all genders synchronizing LF processes more within friendships. Since gender differences start to intensify during this developmental period^77^, future research should investigate gender-specific interaction patterns at the physiological level and explore the distinct characteristics of parasympathetic and sympathetic synchronization. Peer dynamics underlie gender homophily, with children being more likely to identify with and befriend same-gender rather than opposite-gender peers^78^. In the context of peer exclusion, girls show greater liking for female victims than for male victims^79^. Therefore, it is possible that all-female friend dyads may have empathized more with the female main character compared to other dyads. While there were no differences in reported valence or arousal by gender (see SM S4.3), we cannot account for differences in social identification processes. Therefore, future research is needed to better understand the interplay between gender identification, affective processes, and physiological synchrony in the context of peer relations and social exclusion.

In contrast to friendship status, closeness and liking were not significantly related to synchrony. However, closeness and liking were both positively related to friendship status (see Table 2 in SM S4.1). Additional analyses explored potential interaction effects between friendship, liking, closeness, and affective experiences. The results indicated that, within friendships, those who expressed more liking for each other showed decreased LF synchrony, suggesting a potential influence of friendship quality on synchronization in friend dyads (see also^24^). For non-friends, decreased LF synchrony was observed when they felt closer to each other, and when they more accurately estimated each other’s affect. In addition, affective arousal was related to decreased LF synchrony in non-friends but to increased LF synchrony in non-reciprocated friendships. Taken together, these findings point to the interaction effects between relationship variables and affective variables^42,44^, which should be explored in more detail in future research. In addition, future work could investigate additional contexts where people with different relationship types interact. For example, synchrony can also emerge in dyads of strangers^48,80^, and strangers may even synchronize more than friends in some situations, as interactions with strangers require active building of rapport^48^. In order to extend the results of the current study, future work could look at interactions of children who are not acquainted, as compared to those who are acquainted, and those who are friends. A longitudinal follow-up study could further shed light on how different developments in relationship characteristics may bring about changes in synchronization patterns over time^81^.

Beyond the variables of interest, we saw an effect of the task (listening to the story versus discussing the story) on synchrony, with higher HF and LF synchrony during the discussion than during the story. Discussions are characterized by turn-taking, which requires increased attention to others’ subtle cues that indicate changes of speakers^82^. Future research should include measures of attention and engagement during each task, which have been linked to increased synchronization^83,84^. This effect explained more variance than all other predictors, highlighting the importance of selecting an appropriate task to measure synchronization. While both HF and LF synchrony descriptively increased during the story and the discussion compared to the resting baseline, we did not include synchrony measured during the baseline in our analysis. Only half of the group underwent the baseline task together, and some children interacted despite being told to focus on themselves. Therefore, the baseline measure does not constitute a “true” baseline. Best practice would be to provide a separate room for each participant, and to measure the baseline at the same time, which was not feasible due to resource constraints.

While the current study reveals novel findings regarding peer group interactions and their underlying physiological processes, benefiting from a large sample size and naturalistic setting, some additional limitations should be mentioned. The current analyses investigated synchrony on the dyadic level, whereas the experiment was conducted in a group setting. As groups possess specific characteristics beyond dyadic connections^1^, group-level predictors and processes would likely explain additional variance in interactional outcomes. However, even the level of the dyad remains poorly understood, which we felt was a prerequisite for investigating group processes elsewhere. We tried to mitigate this limitation by including a random group effect in the synchrony models, which explained ca. 9% (for HF synchrony) and 2% (for LF synchrony) of additional variance. Future studies should investigate the effects of group size (dyads, triads, and larger groups) on synchronization. Moreover, the role of the individual in peer interactions is not yet well understood, particularly in the context of peer groups. Individuals have a tendency to synchronize more or less across interactions and relationships^85–87^. Some individuals may be “super-synchronizers”^87^ and may synchronize more easily. The influence of individual characteristics should be considered in future research. Furthermore, the discussion task was designed to be naturalistic and elicit authentic responses from participants in order to increase ecological validity. However, this lack of standardization makes it difficult to compare this task between groups or describe individual phases within the task. For example, the specific discussion points may have elicited related moment-to-moment affect, which we did not capture with our measure of affect. As a methodological consideration, there is no consensus on the optimal way to analyze autonomic nervous system synchrony, and cross-wavelet power is far from the only viable analysis method^88^. Here, we base our choice of method on preliminary qualitative comparisons between methodological approaches, taking into account the properties of our data and underlying research question (see^89^). In the future, unified guidelines should be discussed in the field of physiological synchrony research to arrive at an expert consensus^43^.

By measuring physiological synchrony within peer group contexts, we show that synchrony in children’s interactions is shaped by relational factors, such as friendship, and situation-specific affective factors, including affective responses to shared experiences. Tracking moment-to-moment changes in synchrony during social interactions offers a window into the evolving dynamics of peer groups and the interpersonal processes that shape children’s social development^45,81^. Extending synchrony research into late childhood and early adolescence highlights its potential as a meaningful marker of interpersonal connection, moving beyond individual-level measures to reveal how physiological alignment contributes to the formation and maintenance of social relationships.

## Supplementary Information

Supplementary Information.

## Data Availability

Data used in the analysis is available under https://osf.io/tbrh9/?view_only=691e7e9eac814645a424a3ea0ac527ad.
