# Supplementary material for: Cardiac synchrony, peer relationships, and affective experiences in children during group interactions
Source: Sci Rep. 2026 Feb 25;16:7740. doi: 10.1038/s41598-026-41275-y (PMC12949066; doi:10.1038/s41598-026-41275-y)
Supplement: Supplementary file 1 — Supplementary Information. [file 41598_2026_41275_MOESM1_ESM.pdf]

Supplementary Materials

February 18, 2026

S1 Additional information about the study design

S1.1 Summary of the story

The characters in the story were meant to convey different peer roles: victim, bully, helper, or bystander. These peer roles were introduced to the children before they heard the story and defined in the following way:

- *Victims* are children who experience bullying, but do not bully others.
- *Bullies* are children who victimize others, but are not bullied themselves.
- *Helpers* are children who try to help the victim.
- *Bystanders* are children who do not actively bully, but do not help the victim either.

The story depicts (*Yara*), a fifth-grade student new to her class, who initially feels shy but gradually becomes more integrated. A highlight of the school year is a basketball tournament between all fifth-grade classes, which many classmates eagerly anticipate, practicing for weeks and imagining future basketball careers. Yara also trains with her brothers and looks forward to the event. In the final game, she wishes to let the more athletic players take over, but the teacher requires her to stay on the court. At the decisive moment, she misses the ball, is struck on the head, and loses her glasses, which enables the opposing team to score the winning point. She is mocked by (*Remo*), a peer from the opposing team. (*Liam*) briefly defends her but withdraws when he himself becomes the target of ridicule. In the following days, (*Vera and Selma*) escalate the bullying, excluding Yara and making discriminatory remarks about her family's migrant background, while her closest friend (*Nathalie*) distances herself. Yara feels ashamed, overwhelmed, and wishes she could disappear. Some of the other children discuss whether they should stop being mean to Yara, but then disengage morally by blaming the victim and by downplaying the severity of their actions. Not all children agree and some of the peers feel bad about the situation. The story ends with (*Cristian*) objecting to the discriminatory remarks, though his intervention is opposed by the bullies.

S1.2 Discussion questions

The following discussion questions were presented to the children (translated from the original German):

1. Summarize the story in 2-3 sentences (briefly), what is it about? Describe the people involved. What do they do in the story and why?
2. Is Liam's behavior toward Yara fair? Why (not)?
3. Is Nathalie's behavior towards Yara fair? Why (not)?
4. Do the children have any concerns about their behavior towards Yara? (Liam, Cristian, Vera, Selma)
5. How do these children justify their behavior? Is this okay in your opinion or not?
6. Feelings also play an important role in the story. How does Liam feel? How do Vera and Nathalie feel? And Selma?
7. How does Yara feel at school? What could happen to Yara in the worst-case scenario as the story progresses? And in the best-case scenario?
8. Is the way the children behave towards Yara bullying? Why (not)?
9. Is Yara being discriminated against? What does this mean?
10. Are the children biased towards Yara (prejudices)? What do they think of her?

The experimenter presented the questions to the children one at a time. For each question, they read it aloud and then placed a paper with the question in the center of the circle of participants, allowing the children to discuss it independently while stepping back. Once the discussion about a question concluded, the experimenter returned to the group to read the next question.

S2 Additional information about the measures used in the study

S2.1 Measures of affect coherence and empathic accuracy

We define affect coherence as the reversed difference between two individuals' affective responses to a situation. In our study, we used an adapted version of the Affect Grid [1] to measure children's self-reported affect following the story and discussion tasks. To calculate affect coherence in a dyad, we measure the Euclidean distance between two children's Affect Grid values, as illustrated by Figure 1.

In the example, we take a dyad composed of child A and child B. Child A states an affect of valence = 3 (counted from left to right), and arousal = 3

Figure 1: Example for the affect coherence measure calculated using the Affect Grid [1]. Affect coherence is measured as the distance between two children's affect values, subtracted from the maximum value.

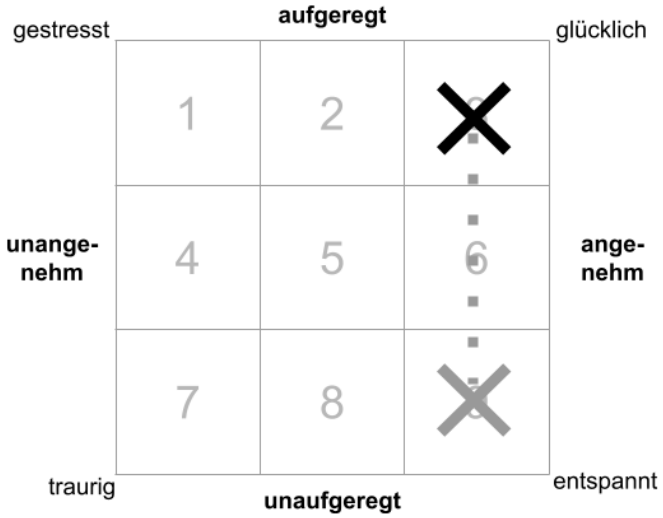

(counted from bottom to top). The affect of Child A is marked in black on the grid. Child B may feel more relaxed, stating an affect of valence = 3, arousal = 1 (marked in dark grey on the grid). The Euclidean distance between the coordinates (3,3) and (3,1) is calculated as follows:

$$\text{dist}_{ab} = \sqrt{(\text{valence}_a - \text{valence}_b)^2 + (\text{arousal}_a - \text{arousal}_b)^2} \tag{1}$$

For easier interpretation, the distance is subtracted from the maximum possible distance ($\text{dist}_{max} \approx 2.83$) to arrive at a quantification of affect coherence:

$$\text{affect coherence}_{ab} = \text{dist}_{max} - \sqrt{(\text{valence}_a - \text{valence}_b)^2 + (\text{arousal}_a - \text{arousal}_b)^2} \tag{2}$$

Thus, the affect coherence value in the example would be:

$$\text{affect coherence}_{ab} = 2.83 - \sqrt{(3-3)^2 + (3-1)^2} \approx 0.83 \tag{3}$$

Empathic accuracy was measured similarly to affect coherence, but resulted in one value per dyad member, instead of a shared value as for affect coherence. Empathic accuracy is typically measured as a rating of the agreement between

Figure 2: Example for the empathic measure calculated using the Affect Grid [1]. Empathic accuracy is defined as the distance between a child's affect values and the estimate of the same child's affect values by another child, subtracted from the maximum value.

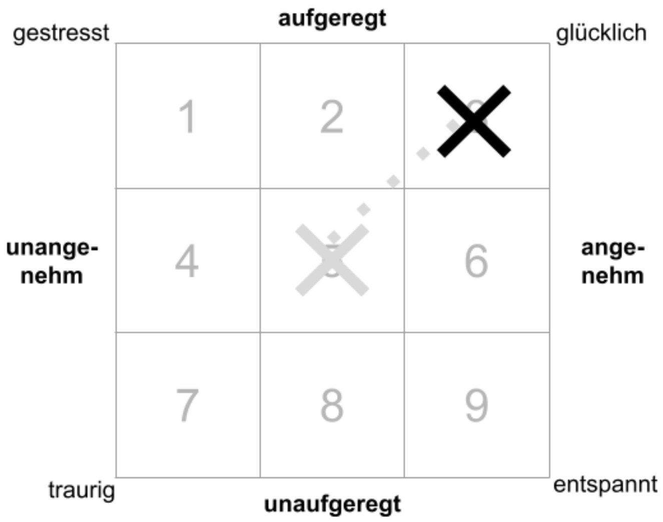

one person's stated emotions and another person's inference of the first person's emotions [2, 3]. Here, we again used the distance between actual and inferred values. In Figure 2, child B may have inferred child A's affect to be at valence = 2, arousal = 2 (light grey cross), whereas child A's actual affect was valence = 3, arousal = 3. Child B's empathic accuracy would thus be characterized similarly to Eq.3:

$$\text{empathic accuracy}_{ab} = 2.83 - \sqrt{(3-2)^2 + (3-2)^2} \approx 1.41 \tag{4}$$

As the equations show, there is no differential weighting of valence or arousal. The underlying model for the Affect Grid is the Circumplex Model of Affect [1, 4], which assumes both dimensions to be independent with no specific weighting of one dimension for the calculation of its score. While the independence of dimensions has been contested [5], following the Circumplex Model, we can see the Affect Grid as a coordinate system, which enables the calculation of affect coherence and empathic accuracy as presented here.

S2.2 Pipeline for calculating heart rate synchrony

Raw R-R intervals were recorded during the experiment and processed in R [6]. Raw R-R data were preprocessed to correct for ectopic beats or gaps in the recording. We used an automated pre-processing script, in which values deviating from the previous value by $\geq$ 30% were defined as outliers [see 7]. After outliers were removed, the data needed to be interpolated to avoid gaps in the time series. When a gap was $\geq$ 2 seconds, we interpolated missing data using surrounding values. For participants with $\geq$ 10% outliers, all heart rate data were excluded from the analysis. From the outlier-corrected R-R interval data, we interpolated one heart rate value per second using the RHRV package [8]. Figure 3 provides an example of the raw versus outlier-corrected heart rate values of a child, and shows the interpolation of irregularly sampled R-R interval data to regularly sampled heart rate data with a sampling frequency of 1 Hz.

For each dyad, synchrony was calculated for all time points where both dyad members had good-quality heart rate data. For cross-wavelet power analysis, complete time series are necessary. For some children, large gaps in the recordings were filled with interpolated artificial data to allow synchrony calculation to be performed. These time points were later excluded from the synchrony analysis, which resulted in a variable number of valid synchrony time series points. Data missing in a time series of a dyad can be handled using multilevel modeling.

Cross-wavelet power analysis was performed using the WaveletComp package [9]. Cross-wavelet power analysis is a time-frequency-based analysis. While synchrony is quantified with a high temporal resolution (1 Hz, i.e., the measurement frequency of the heart rate data), there is an additional frequency resolution. In heart rate analysis, frequency information can identify underlying processes – specifically, high-frequency changes in heart rate (0.15-0.4 Hz) indicate parasympathetic activation [10, 11]. In heart rate synchrony analysis,

Figure 3: Example to show the preprocessing of heart rate data. In the first step, outliers were removed (top). In the second step, non-interpolated R-R intervals were interpolated to result in one heart rate value per second.

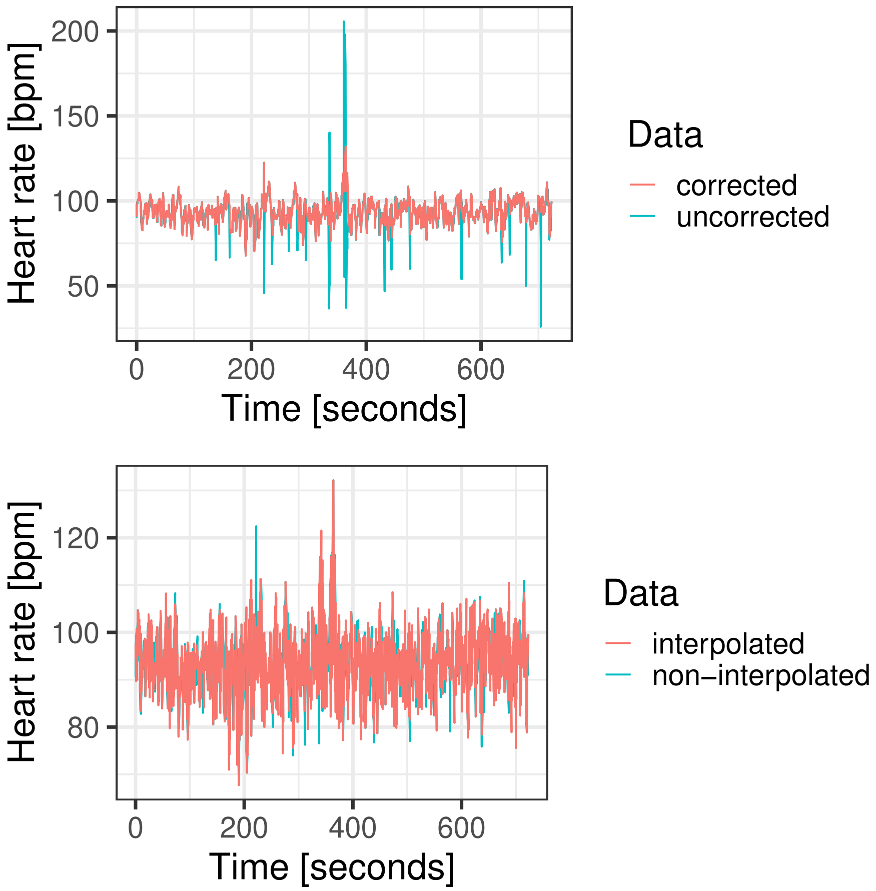

Figure 4: Example results for two different dyads. Dyad one includes the exemplary data shown in Figure 3. The panels on the left show the heart rate trajectories of two participants each. The panels on the right show the corresponding synchrony results.

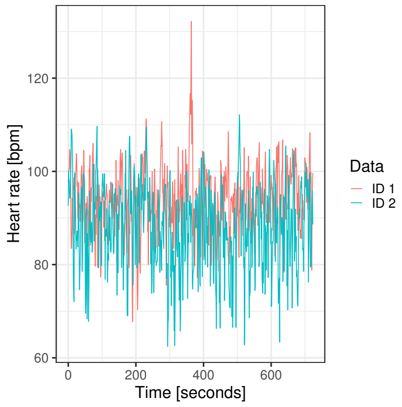

a: Example showing the heart rate trajectories of dyad 1, consisting of participants 1 and 2. The x-axis shows the time in seconds.

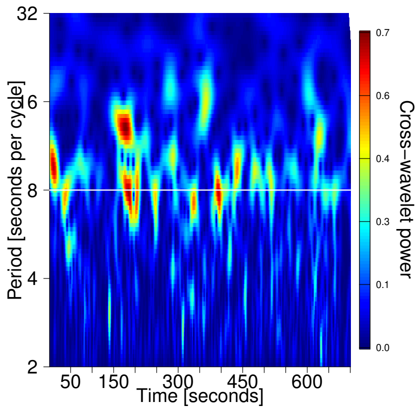

b: Resulting cross-wavelet power plot for dyad 1. The x-axis shows the time in seconds, indicating the changes in heart rate that correspond with instances of high synchrony. The white line indicates the division into high compared to low frequencies.

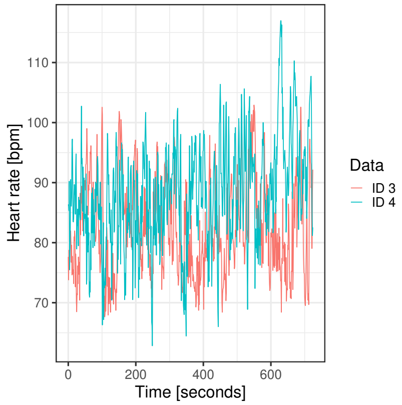

c: Example showing the heart rate trajectories of dyad 2 (participants 3 and 4).

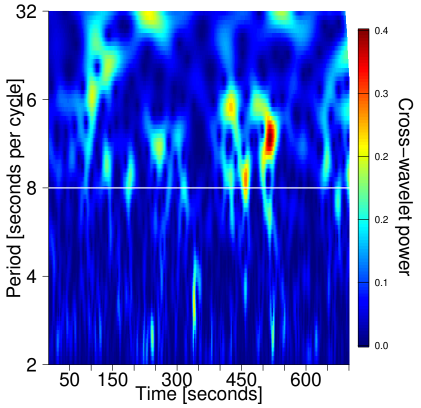

d: Resulting cross-wavelet power plot for dyad 2.

Figure 5: Maximum cross-wavelet power values over time for example dyads 1 and 2. The solid line depicts high-frequency CWP, the dashed line low-frequency CWP. The x-axis shows the time since the beginning of the experimental phase to enable comparisons across dyads.

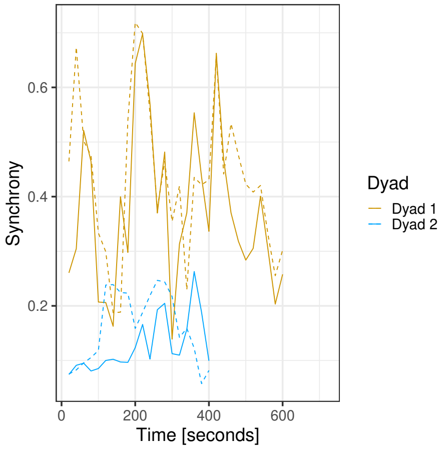

high-frequency bands can similarly indicate parasympathetic synchronization [12]. Lower-frequency components of heart rate trajectories are driven by both sympathetic and parasympathetic activation [10]. As parasympathetic and sympathetic synchronization can diverge in its associations with other variables [13], and parasympathetic processes are related to emotional processing and social aspects of emotions [14, 15], we differentiate specifically parasympathetic synchrony from mixed parasympathetic and sympathetic synchrony.

Figure 4 shows the heart rate data of two example dyads and the respective synchrony analysis results. The cross-wavelet power plots consist of *cross-wavelet power coefficients* (≥ 0) per time point and frequency band, with higher values indicating increased synchrony. To perform a cross-wavelet power analysis, a *wavelet transform* is first applied to each dyad member's heart rate time series. This process, through the convolution with a kernel (here, the Morlet wavelet function; [9]), provides the frequency information about each person's heart rate time series. For example, a heart rate trajectory may be characterized by fast oscillations around a more stable mean (more high-frequency power), or smaller moment-to-moment changes but more pronounced changes in trend (more low-frequency power). Applying the wavelet transform quantifies the relative power in each frequency band across time. In a second step, the *cross-wavelet power analysis* quantifies the similarities between two dyad members' wavelet-transformed heart rate trajectories. Higher values thus indicate more similarity, including in-phase (changes in the same direction) and anti-phase (changes in opposite directions) synchronous trajectories, and shifted synchrony (one person's heart rate values change before the other person's).

The two-dimensional (time x frequency) result is then aggregated into a one-

dimensional synchrony time series. Here, no guidelines for the aggregation are available yet. To avoid both loss of information and redundancy, we extracted the maximum value for each 20-second segment across each task in the experiment, thus preserving moments of high synchrony. The choice of 20-second intervals was used as a compromise between sufficient temporal resolution and redundancy. While the temporal resolution could be increased, subsequent values are autocorrelated due to the nature of the wavelet function. As this aggregation is only used after synchrony results have been determined, there is no impact of the aggregation on calculating the synchrony values themselves. As described above, we differentiate between parasympathetic and mixed sympathetic and parasympathetic types of synchrony, and extract the maximum for both frequency bands (see Figure 5).

S3 Statistical analysis

S3.1 Building the multilevel models

To account for the nested structure of our data, we used multilevel models including random effects. To build the models, we followed the procedure described by [16].

For the analysis of HF HRV, HRV values were measured at different time points (level 1), nested in different experimental phases (level 2), nested in individuals (level 3), nested in groups (level 4). For the model notation, see the section *Model notations*. We first calculated intra-class coefficients (ICCs) for individuals and groups, following the procedure described in [16]:

$$\mathrm{ICC}_i = \frac{\tau_i + \tau_g}{\tau_i + \tau_g + \sigma^2} \tag{5}$$

where τ_i is the between-group variance for individuals, τ_g is the between-group variance for groups, and σ^2 is the residual variance, following the notation by [16]. ICC_i denotes the ICC for individuals, which was 61.96% in our sample, thus justifying the use of random effects modeling. The level-4 (group-level) ICC was calculated as:

$$\mathrm{ICC}_g = \frac{\tau_g}{\tau_d + \tau_g + \sigma^2} \tag{6}$$

ICC_g was 15.75% in our sample.

In the next step, a time variable is added to the random intercept HF HRV model to assess the temporal development of the dependent variable. Table 1 (top) shows the model comparisons to establish random effects and fixed time effects for HF HRV. Adding a fixed effect for time and phase, and a random slope for time, significantly improved the model fit. While adding a random slope across experimental phases resulted in a significant likelihood ratio test coefficient, model quality indicators showed a worse model fit. Thus, a random slope across phases was not included in the model. Adding an autocorrelative (AR1) structure resulted in a model that did not compile; however, it improved

model fit in the full model. Based on the results, the model for HF HRV included random intercepts per individual, fixed effects for time during each experimental phase, experimental phase, a random slope across time and phase per individual, and an AR1 structure.

For the analysis of synchrony, we measured HF and LF synchrony at different time points (level 1), which were nested in experimental phases (level 2), which were nested in dyad (level 3), which was nested in group (level 4; see section *Model notations*). We calculated the level-3 (dyad-level) ICC as:

$$\mathrm{ICC}_d = \frac{\tau_d + \tau_g}{\tau_d + \tau_g + \sigma^2} \tag{7}$$

where τ_d is the between-group variance for dyads, τ_g is the between-group variance for groups, and σ^2 is the residual variance, following the notation by [16]. The level-4 (group-level) ICC was calculated as:

$$\mathrm{ICC}_g = \frac{\tau_g}{\tau_d + \tau_g + \sigma^2} \tag{8}$$

For HF synchrony, we found ICC_d = 15.26%, and ICC_g = 8.90%. For LF synchrony, we found ICC_d = 7.75% and ICC_g = 2.13%. This indicates that random effects on both levels can explain some variance in HF and LF synchrony, and random intercepts for dyad and group were included in both models.

Table 1 shows the model comparisons for models predicting HF synchrony (middle) and LF synchrony (bottom). For both synchrony measures, adding random intercepts for dyads and groups improved the model fit. A fixed effect for time within phases was added for LF synchrony, but not HF synchrony. Adding the experimental phase resulted in a better model fit for both HF and LF synchrony models. A random slope for time was added for the HF synchrony model, but not the LF synchrony model. Random slopes across phases did not explain additional variance in either model. Both models additionally included an AR1 structure.

S3.2 Addition of dyadic variables

In hypothesis 2, we averaged the independent variables ”closeness” and ”liking” scores measured before and after the experiment. This strategy was chosen to reduce the number of variables in the model to avoid overfitting. Further, we used the sum score of the averaged individual scores for each dyad, for ”closeness”, ”liking”, ”affective arousal”, ”affective valence”, and ”empathic accuracy”. This enables testing the connection between synchrony and the total amount of the respective variable in the dyad.

S3.3 Model notations

The model for testing hypothesis 1, i.e., the connection between heart rate variability (HRV) and individual affect, we formulated the model as (notation

Table 1: Stepwise model comparisons to build a base model including random effects, for high-frequency heart rate variability (HF HRV), high-frequency (HF) synchrony, and low-frequency (LF) synchrony.

HF HRV		
Model including	Likelihood ratio	p-value
Random intercepts for individual and group	2253.52	$< .001$
Fixed effect for time	33.20	$< .001$
Fixed effect for phase	13.38	.001
Random slope for time	11.61	.003
Random slope for phase	791.98	$< .001$[a]
Autocorrelative structure	[b]	
**HF synchrony**		
Model including	Likelihood ratio	p-value
Random intercepts for dyad and group	1078.43	$< .001$
Fixed effect for time	< 0.01	.976
Fixed effect for phase	182.00	$< .001$
Random slope for time	45.22	$< .001$
Random slope for phase	> 0.01	1
Autocorrelative structure	1535.57	$< .001$
**LF synchrony**		
Model including	Likelihood ratio	p-value
Random intercepts for dyad and group	550.56	$< .001$
Fixed effect for time	13.52	$< .001$
Fixed effect for phase	879.09	$< .001$
Random slope for time	> 0.01	1
Random slope for phase	> 0.01	1
Autocorrelative structure	3917.00	$< .001$

Note: [a] despite the significant result, model fit was worse when a random slope for phase was included.
[b] This model did not compile, but comparisons of the full model indicate a better fit when using an autocorrelative structure.
Phase was added as a dummy-coded variable with the reference "resting baseline" for the HF HRV model, and with the reference "story" for the HF and LF synchrony models.

adapted from [17]):

$$\begin{aligned}\text{HF HRV}_{pti} = {} & \beta_{00} + \beta_{10} * \text{phase}_{pi} + \beta_{20} * \text{time}_{ti} + \\ & \beta_{30} * \text{Gender}_i + \beta_{30} * \text{Age}_i + \beta_{30} * \text{BMI}_i + \\ & \beta_{40} * \text{Arousal}_i + \beta_{50} * \text{Valence}_i + \\ & \beta_{60} * \text{Contentness}_i + \beta_{70} * \text{Safety}_i + \beta_{80} * \text{Stress}_i + \\ & U_{0i} + U_{1i} * \text{time}_{ti} + e_{ti}\end{aligned} \tag{9}$$

HF HRV is measured for individual i ($i = 1, ..., N$) at time point t ($t = 30, 90, ..., T$) in experimental phase p ($p = 1, 2, 3$). The level-1 predictor was the time in seconds during each phase (measured in intervals of 60 seconds). Time was nested within the experimental phase on level 2. Level-3 predictors were the affect-related variables and covariates. β-values represent regression coefficients. U_{0i} denotes random intercepts for each individual i, and U_{1i} and U_{2i} represent random slopes, where $U = \mathcal{N}(0, \sigma^2_{U0})$. The random error is given by $e_{ti} = \mathcal{N}(0, \sigma^2_e)$.

For testing hypothesis 2, i.e., the relation between synchrony and relational and affect-related variables, we formulated the model analogously as:

$$\begin{aligned}\text{HF/LF CWP}_{ptd} = {} & \beta_{00} + \beta_{10} * \text{phase}_{pd} + \beta_{20} * \text{time}_{td} + \\ & \beta_{30} * \text{Gender combination}_d + \beta_{40} * \text{Friendship status}_d + \\ & \beta_{50} * \text{Closeness [sum]}_d + \beta_{60} * \text{Liking [sum]}_d + \\ & \beta_{70} * \text{Affect coherence}_d + \beta_{80} * \text{Arousal [sum]}_d + \\ & \beta_{90} * \text{Valence [sum]}_d + \beta_{100} * \text{Empathic accuracy[sum]}_d + \\ & U_{0d} + U_{1d} * \text{time}_{td} + U_{2d} * \text{phase}_{pd} + V_{0g} + e_{td}\end{aligned} \tag{10}$$

Synchrony (HF or LF cross-wavelet power) was measured for dyad d ($d = 1, ..., N$) at time point t ($t = 20, 40, ..., T$) in experimental phase p ($p = 1, 2$). On the lowest level, synchrony was predicted by time in seconds during each phase (measured in intervals of 20 seconds). Time was nested within the level-2 predictor experimental phase. Level-3 predictors were dyads, with measures of relational and affective variables. Dyads were additionally nested in groups g ($g = 1, ...M$). U_{0d} denotes the random intercept for each dyad d, with U_{1d} and U_{2d} representing random slopes, where $U = \mathcal{N}(0, \sigma^2_{U0})$. V_{0g} represents the random intercept for each group, $V = \mathcal{N}(0, \sigma^2_{V0})$. Note that the variable time was not included for HF synchrony (see S3.1).

S3.4 Deviations from preregistration

We had preregistered the study prior to the data analysis (`https://osf.io/tbrh9/?view_only=691e7e9eac814645a424a3ea0ac527ad`). However, we deviated from the preregistration in some instances due to novel insights into the data processing and some unforeseen characteristics of the data. Specifically, we chose different approaches in the following points:

- We aimed to analyze synchrony in reference to the specific events in the story (specifically, dividing the story into < 200 seconds (before an event in the story where the protagonist is excluded) and > 200 seconds (after the exclusion event). However, for some groups, the beginning of the story had to be repeated, or good-quality data is only available for the last minutes. Therefore, an overall measure seemed more appropriate than this division.

- We planned to interpolate heart rate data with a sampling frequency of 4 Hz. However, we saw in other projects that the sampling frequency of 1 Hz does not substantially change the resulting heart rate trajectory, and has the advantage of a sparser representation of data.

- We planned to include group norm measures, but found this to be beyond the scope of the current analysis, and the measure will be reported elsewhere.

- Smile synchronization data will be reported elsewhere (manuscript in preparation), as it would be beyond the scope of the current analysis. The two different modalities are on different time scales, require different analytical procedures, and cannot directly be linked within our current approach.

- We planned to examine changes in affective experiences in connection with high-frequency heart rate variability. However, due to an error in the questionnaire, we did not assess affective experiences before the task.

- We did not assess synchronization during the baseline, as a) children in the same room may have influenced each other during the baseline, and b) reviews of previous projects advised against the calculation of synchronization for artificial dyads.

- We planned to account for global motion energy, but could not properly align the data with heart rate data.

- Changes in synchrony over time were only implicitly tested in our model and not related to the changes in perceived relationship characteristics explicitly. Based on more detailed reviews of possible methodologies, we believe that answering this question would require a different analytic approach and decided that adding this would be too excessive given the scope of the current analyses.

S4 Additional results

S4.1 Correlation between predictor variables

Table 2 shows the Pearson's correlation coefficients between the predictor variables used in the model for hypothesis 2 ($N = 243$). Within the relationship-related variables, we find a high correlation coefficient between friendship status

Table 2: Person's correlation coefficients for predictor variables.

	1	2	3	4	5	6	7
1. Friendship status	1	0.55*	0.77*	0.08	0.07	0.00	0.07
2. Liking sum	0.55*	1	0.76*	0.05	0.09	0.08	0.13*
3. Closeness sum	0.77*	0.76*	1	0.09	0.07	0.05	0.15*
4. Affect coherence	0.08	0.05	0.09	1	0.39*	-0.14*	0.76*
5. Valence sum	0.07	0.09	0.07	0.39*	1	-0.02	0.25*
6. Arousal sum	0.00	0.08	0.05	-0.14*	-0.02	1	-0.06
7. Empathic accuracy	0.07	0.13*	0.15*	0.76*	0.25*	-0.06	1

(re-coded as an ordinal variable with 1 for no friendship, 2 for one-sided friendship, and 3 for mutual friendship) and closeness, as well as closeness and liking. While the variance inflation factors (VIF) in both hypothesis 2 models were relatively low (see the section *Model assumptions*), the VIF for closeness was > 2, indicating some multicollinearity. Thus, variance in synchrony explained by closeness may already be accounted for by variance explained by friendship status, obfuscating the effect of closeness on synchrony.

For affect-related variables, there was a high correlation coefficient for affect coherence and empathic accuracy. The connection between similar affective states and empathic accuracy has been previously reported in therapist-client dyads [18], and could be explained through the connection between emotional contagion / emotional synchrony and empathic processes [19]. Interestingly, the exploratory results (see manuscript section *Results – Heart rate synchrony, relationships, and affective experiences*) indicate different effects of empathic accuracy depending on friendship status.

S4.2 Assumptions testing and model fit

Linear mixed effects models have several assumptions, which we tested for each model. The method assumes the normality of residuals, the independence and homoscedasticity of residuals, and low multicollinearity. We present the normal quantile-quantile (Q-Q) plots to check the normality of residuals, and show the residuals plotted against fitted values to assess homoscedasticity and independence of residuals. We also assess variance inflation factors (VIFs) for each predictor variable to check for multicollinearity, where we report the generalized VIF adjusted for the degrees of freedom for each term ($GVIF^{1/(2xdf)}$) [see 20].

The fixed effects in the model for HF synchrony could explain 3.2% of variance. Figure 6 shows the Q-Q plot and residuals for the HF synchrony model. The plots indicate that higher synchrony values cannot be as accurately predicted as lower and medium-high synchrony values. Thus, the predictor variables included in the model cannot explain those instances where synchrony was particularly increased. Additional factors that may be inherent to the dyadic dynamics might contribute to instances of high HF synchronization. Table 3 shows that the VIFs for HF synchrony did not exceed one by a large amount,

indicating sufficiently small multicollinearity between factors.

For LF synchrony, the main model's fixed effects could explain 11.5% of the variance in the dependent variable. Figure 7 shows that, similarly to HF synchrony, LF synchrony is better predicted by the model for low and medium values than for higher LF synchrony values. VIFs for LF synchrony predictors are similar to those for HF synchrony predictors, indicating sufficiently low multicollinearity.

Table 3: Variance inflation factors (GVIF) adjusted for degrees of freedom (df) for predictors in different models to assess multicollinearity. A GVIF close to one indicates no multicollinearity.

HF synchrony	
Variable	$GVIF^{1/(2xdf)}$
Task	1.00
Gender	1.20
Friendship status	1.30
Closeness (sum)	2.10
Liking (sum)	1.59
Affect coherence	1.72
Affective arousal (sum)	1.02
Affective valence (sum)	1.13
Empathic accuracy	1.64
LF synchrony	
Variable	$GVIF^{1/(2xdf)}$
Task	1.00
Time	1.00
Gender	1.20
Friendship status	1.29
Closeness (sum)	2.08
Liking (sum)	1.59
Affect coherence	1.74
Affective arousal (sum)	1.02
Affective valence (sum)	1.13
Empathic accuracy	1.65

S4.3 Exploratory analyses

As friendship status was related to LF synchrony outcomes (see *Results – Heart rate synchrony, relationships, and affective experiences*), we conducted an exploratory analysis including the interaction between friendship and other relationship quality variables, as well as between friendship and affect-related variables. Table 4 shows the regression coefficients and significance of the predictors in the exploratory models for HF synchrony. Results for LF synchrony are shown in Table 5.

Figure 6: Model fit indicators for the HF synchrony model: Q-Q plot (top), and residuals plotted against fitted values (bottom).

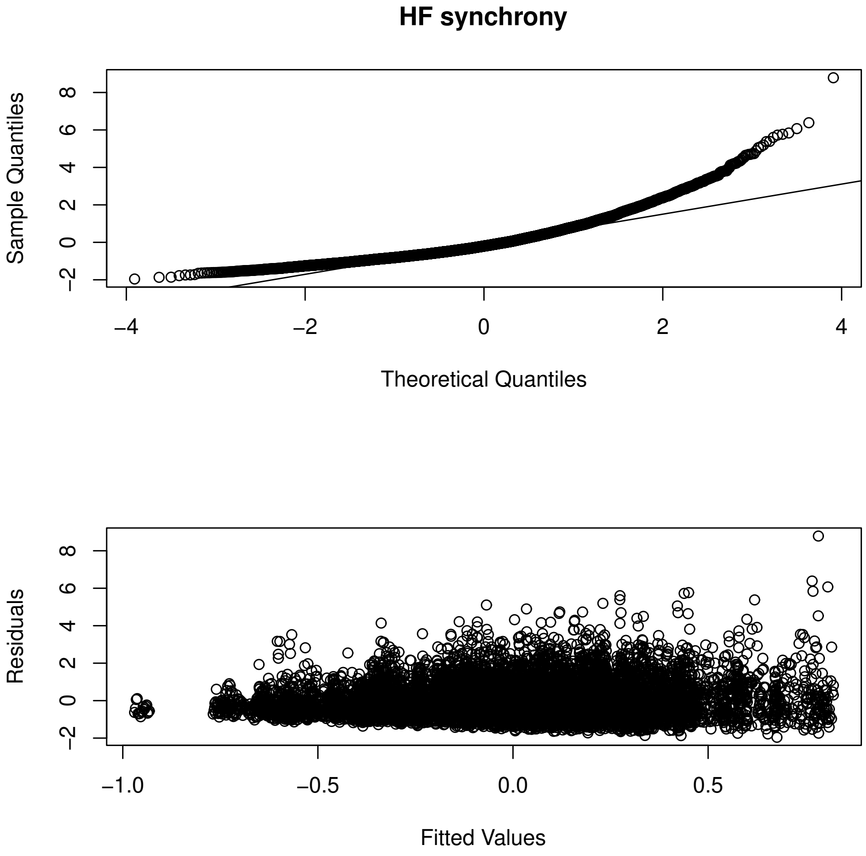

Figure 7: Model fit indicators for the LF synchrony model: Q-Q plot (top), and residuals plotted against fitted values (bottom).

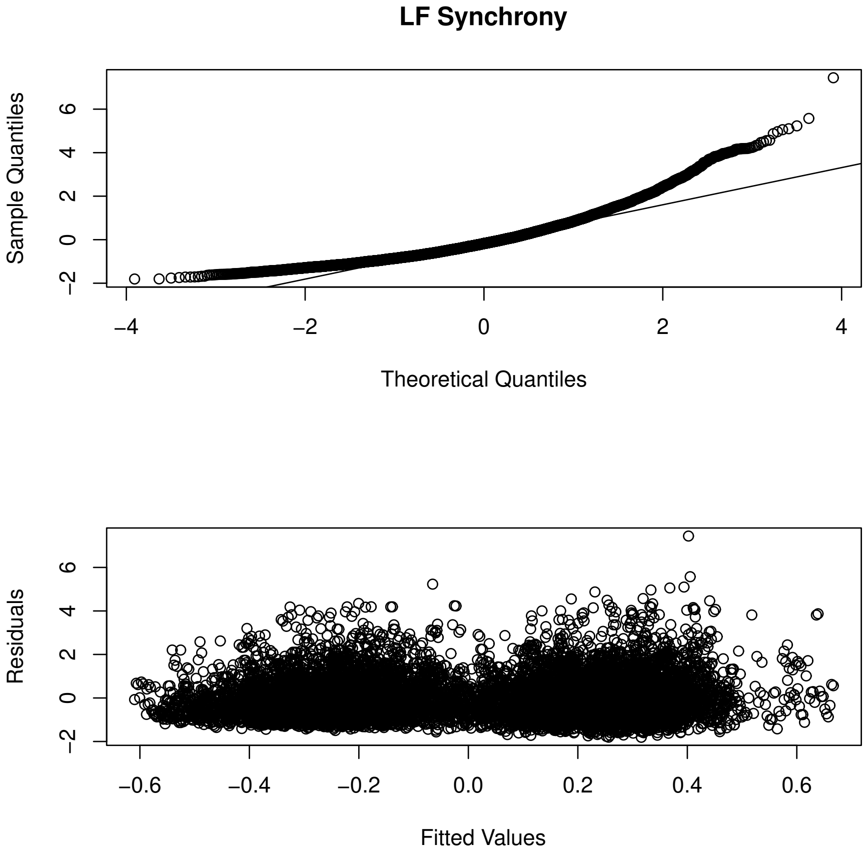

In the exploratory analysis, we saw an interaction between friendship status and gender influencing HF synchrony. The story included a female protagonist, which may have influenced children's emotional reaction depending on gender. To test this possibility, we compared affective valence and arousal between boys and girls. We found no significant difference in valence or arousal. Average valence was 2.69 for girls (SD = 0.61), and 2.79 for boys (SD = 0.46; t(171.65) = -1.23, p = .220). Girls reported a mean arousal of 1.88 (SD = 0.76), compared to a mean arousal of 1.99 for boys (SD = 0.78; t(179.36) = -0.94, p = .350).

Both types of synchrony were higher during the discussion compared to the story. This may be related to an overall increased activation. To assess the difference in HF HRV between story and discussion, we adjusted the model for HF HRV by including only these phases, and removing any predictors other than time during each phase, and experimental phase. We found no significant difference in HF HRV between the story and the discussion phase (see Table 6), indicating that differences in synchrony between the phases are not simply an effect of increased overall parasympathetic activation.

References

[1] Russell, J.A., Weiss, A., and Mendelsohn, G.A. Affect Grid: A Single-Item Scale of Pleasure and Arousal. *Journal of Personality and Social Psychology* 57.3 (1989), 493–502.

[2] Fujiwara, K. and Daibo, I. Empathic accuracy and interpersonal coordination: behavior matching can enhance accuracy but interactional synchrony may not. *The Journal of Social Psychology* 162.1 (2022), 71–88. `https://doi.org/10.1080/00224545.2021.1983509`.

[3] Ta, V.P. and Ickes, W. "Empathic accuracy". *The Routledge Handbook of Philosophy of Empathy.* 1st. eBook. Routledge, 2017, 353–363.

[4] Russell, J.A. A circumplex model of affect.. *Journal of Personality and Social Psychology* 39.6 (1980), 1161–1178. `https://doi.org/10.1037/h0077714`.

[5] Rubin, D.C. and Talarico, J.M. A comparison of dimensional models of emotion: Evidence from emotions, prototypical events, autobiographical memories, and words. *Memory* 17.8 (2009), 802–808. `https://doi.org/10.1080/09658210903130764`.

[6] R Core Team. *R: A Language and Environment for Statistical Computing.* R Foundation for Statistical Computing. Vienna, Austria, 2024.

[7] Kemper, K.J., Hamilton, C., and Atkinson, M. Heart Rate Variability: Impact of Differences in Outlier Identification and Management Strategies on Common Measures in Three Clinical Populations. *Pediatric Research* 62.3 (2007), 337–342. `https://doi.org/10.1203/PDR.0b013e318123fbcc`.

[8] Rodriguez-Linares, L., Vila, X., Lado, M.J., Mendez, A., Otero, A., and Garcia, C.A. *RHRV: Heart Rate Variability Analysis of ECG Data.* R package version 5.0.0. 2024. `https://doi.org/10.32614/CRAN.package.RHRV`.

[9] Roesch, A. and Schmidbauer, H. *WaveletComp: Computational Wavelet Analysis.* R package version 1.2. 2025. `https://doi.org/10.32614/CRAN.package.WaveletComp`.

[10] Quigley, K.S., Gianaros, P.J., Norman, G.J., Jennings, J.R., Berntson, G.G., and De Geus, E.J.C. Publication guidelines for human heart rate and heart rate variability studies in psychophysiology—Part 1: Physiological underpinnings and foundations of measurement. *Psychophysiology* 61.9 (2024), e14604. `https://doi.org/10.1111/psyp.14604`.

[11] Laborde, S., Mosley, E., and Thayer, J.F. Heart Rate Variability and Cardiac Vagal Tone in Psychophysiological Research – Recommendations for Experiment Planning, Data Analysis, and Data Reporting. en. *Frontiers in Psychology* 08 (2017). `https://doi.org/10.3389/fpsyg.2017.00213`.

[12] Wienhold, S. et al. The relationship of early life adversity and physiological synchrony within the therapeutic triad in horse-assisted therapy. en. *Journal of Neural Transmission* (2025). https://doi.org/10.1007/s00702-025-02947-7.

[13] Danyluck, C. and Page-Gould, E. Social and Physiological Context can Affect the Meaning of Physiological Synchrony. *Scientific Reports* 9.1 (2019), 8222. https://doi.org/10.1038/s41598-019-44667-5.

[14] Thayer, J.F. and Lane, R.D. A model of neurovisceral integration in emotion regulation and dysregulation. *Journal of Affective Disorders* 61.3 (2000), 201–216. https://doi.org/10.1016/S0165-0327(00)00338-4.

[15] Porges, S.W. Social Engagement and Attachment: A Phylogenetic Perspective. *Annals of the New York Academy of Sciences* 1008.1 (2003), 31–47. https://doi.org/10.1196/annals.1301.004.

[16] Bliese, P.D. and Ployhart, R.E. Growth Modeling Using Random Coefficient Models: Model Building, Testing, and Illustrations. *Organizational Research Methods* 5.4 (2002), 362–387. https://doi.org/10.1177/109442802237116.

[17] Finch, W.H. and Bolin, J.E. Multilevel modeling using Mplus. *Multilevel Modeling Using Mplus* (2017), 1–321. https://doi.org/10.1201/9781315165882.

[18] Atzil-Slonim, D. et al. Therapists' empathic accuracy toward their clients' emotions.. *Journal of Consulting and Clinical Psychology* 87.1 (2019), 33–45. https://doi.org/10.1037/ccp0000354.

[19] Prochazkova, E. and Kret, M.E. Connecting minds and sharing emotions through mimicry: A neurocognitive model of emotional contagion. *Neuroscience & Biobehavioral Reviews* 80 (2017), 99–114. https://doi.org/10.1016/j.neubiorev.2017.05.013.

[20] Fox, J. and Weisberg, S. *An R Companion to Applied Regression.* Third. Thousand Oaks CA: Sage, 2019.

Table 4: Regression coefficients, 95% confidence intervals, and p-values for the HF synchrony model including exploratory interactions between friendship status and relational, affective, and control variables.

HF synchrony			
Variable	Estimate	95% CI [lower limit, upper limit]	p-value
(Intercept)	0.26	[-0.42,0.95]	.451
Task: discussion	0.22	[0.17,0.27]	$<$ .001*
Gender: female & female	-0.37	[-0.55,-0.19]	$<$ .001*
Gender: male & male	0.12	[-0.08,0.33]	.244
One-sided friendship	-0.75	[-2.75,1.26]	.465
Mutual friendship	-0.15	[-1.59,1.3]	.841
Non-friends : Closeness (sum)	0.00	[-0.04,0.05]	.974
One-sided friendship : Closeness (sum)	-0.05	[-0.13,0.03]	.225
Mutual friendship : Closeness (sum)	-0.02	[-0.07,0.04]	.531
Non-friends : Liking (sum)	0.03	[-0.08,0.13]	.616
One-sided friendship : Liking (sum)	0.03	[-0.22,0.27]	.827
Mutual friendship : Liking (sum)	0.02	[-0.16,0.21]	.801
Non-friends : Affect coherence	0.10	[-0.04,0.24]	.161
One-sided friendship : Affect coherence	0.03	[-0.24,0.3]	.834
Mutual friendship : Affect coherence	-0.09	[-0.31,0.13]	.418
Non-friends : Affective arousal (sum)	0.00	[-0.06,0.05]	.898
One-sided friendship : Affective arousal (sum)	0.07	[-0.07,0.22]	.309
Mutual friendship : Affective arousal (sum)	0.03	[-0.05,0.12]	.437
Non-friends : Affective valence (sum)	-0.08	[-0.18,0.02]	.113
One-sided friendship: Affective valence (sum)	-0.05	[-0.25,0.16]	.644
Mutual friendship : Affective valence (sum)	-0.10	[-0.25,0.04]	.160
Non-friends : Empathic accuracy (sum)	-0.05	[-0.13,0.03]	.215
One-sided friendship : Empathic accuracy (sum)	0.05	[-0.11,0.21]	.566
Mutual friendship : Empathic accuracy (sum)	0.06	[-0.07,0.19]	.391
One-sided friendship : Gender: female & female	0.61	[0.05,1.16]	.032*
One-sided friendship : Gender: male & male	0.06	[-0.35,0.48]	.765
Mutual friendship : Gender: female & female	0.53	[0.06,1.01]	.027*
Mutual friendship : Gender: male & male	0.14	[-0.35,0.63]	.581

Note: * indicates $p < 0.05$. The variable "Task" was dummy-coded with the story as a reference condition. "Gender" was dummy-coded with the combination "male & female" as a reference condition.

Table 5: Regression coefficients, 95% confidence intervals, and p-values for the LF synchrony model including exploratory interactions between friendship status and relational, affective, and control variables.

LF synchrony			
Variable	Estimate	95% CI [lower limit, upper limit]	p-value
(Intercept)	-0.36	[-0.88,0.15]	.171
Task: discussion	0.50	[0.43,0.56]	< .001*
Time	0.00	[-0,0]	.069
Gender: female & female	-0.17	[-0.31,-0.04]	.014*
Gender: male & male	0.04	[-0.12,0.2]	.597
One-sided friendship	0.01	[-1.5,1.52]	.989
Mutual friendship	0.76	[-0.32,1.84]	.169
Non-friends : Closeness (sum)	-0.05	[-0.08,-0.01]	.008*
One-sided friendship : Closeness (sum)	0.00	[-0.06,0.06]	.917
Mutual friendship : Closeness (sum)	0.00	[-0.04,0.05]	.831
Non-friends : Liking (sum)	0.07	[0,0.15]	.061
One-sided friendship : Liking (sum)	-0.09	[-0.28,0.1]	.344
Mutual friendship : Liking (sum)	-0.15	[-0.29,-0.02]	.029*
Non-friends : Affect coherence	0.08	[-0.03,0.19]	.147
One-sided friendship : Affect coherence	-0.02	[-0.23,0.18]	.816
Mutual friendship : Affect coherence	-0.15	[-0.32,0.02]	.074
Non-friends : Affective arousal (sum)	-0.06	[-0.1,-0.02]	.005*
One-sided friendship : Affective arousal (sum)	0.12	[0.02,0.23]	.025*
Mutual friendship : Affective arousal (sum)	0.03	[-0.04,0.09]	.392
Non-friends : Affective valence (sum)	0.02	[-0.05,0.1]	.564
One-sided friendship: Affective valence (sum)	0.01	[-0.14,0.17]	.851
Mutual friendship : Affective valence (sum)	0.05	[-0.05,0.16]	.315
Non-friends : Empathic accuracy (sum)	-0.07	[-0.13,-0.01]	.030*
One-sided friendship : Empathic accuracy (sum)	0.05	[-0.07,0.17]	.451
Mutual friendship : Empathic accuracy (sum)	0.06	[-0.04,0.16]	.243
One-sided friendship : Gender: female & female	0.00	[-0.41,0.41]	.997
One-sided friendship : Gender: male & male	-0.14	[-0.45,0.17]	.373
Mutual friendship : Gender: female & female	0.20	[-0.15,0.55]	.256
Mutual friendship : Gender: male & male	-0.01	[-0.39,0.36]	.940

Note: * indicates $p < 0.05$. The variable "Task" was dummy-coded with the story as a reference condition. "Gender" was dummy-coded with the combination "male & female" as a reference condition.

Table 6: Exploratory analysis of differences in absolute HF HRV during the story compared to the discussion phase. Regression coefficients, 95% confidence intervals, and p-values.

Variable	Estimate	95% CI [lower limit, upper limit]	p-value
(Intercept)	5.45	[5.25,5.65]	$< .001$*
Task: discussion	-0.07	[-0.15,0.02]	.123
Time	< 0.01	[-0,0]	.054

Note: * indicates $p < 0.05$. The variable "Task" was dummy-coded with the story as a reference condition.
